# Clinicopathological factors influencing the number of stages of Mohs surgery for basal cell carcinoma^☆^

**DOI:** 10.1016/j.abd.2021.08.007

**Published:** 2022-04-02

**Authors:** Joana Calvão, André Pinho, Ana Brinca, Ricardo Vieira

**Affiliations:** a Dermatovenereology Department, Coimbra University Hospital, Coimbra, Portugal; b Faculty of Medicine, Coimbra University Hospital, Coimbra, Portugal

**Keywords:** Carcinoma, basal cell, Dermatology, Mohs surgery

## Abstract

**Background:**

Mohs Micrographic Surgery (MMS) is commonly used to treat high-risk basal cell carcinoma (BCC).

**Objectives:**

Correlate clinicopathologic preoperative features with the number of MMS stages (primary endpoint) and margins (secondary endpoint) required for BCC complete excision.

**Methods:**

We retrospectively analyzed BCCs treated by MMS in a 2-year period at the study’s institution. Variables studied included the patient gender, age, immune status, lesion size, location, if it was a primary, recurrent, or persistent tumor, histopathologic characteristics, number of surgical stages, and amount of tissue excised.

**Results:**

116 BCCs were included. The majority (61.2%, n = 71) required a single-stage surgery for complete clearance, requiring a final margins of 3.11 ± 2.35 mm. Statistically significant differences between locations in different high-risk areas (periocular, perioral, nose, ear) and the number of MMS stages required for complete excision (p = 0.025) were found, with periocular tumours requiring the highest mean of stages (2.29 ± 0.95). An aggressive histopathology significantly influenced the number of MMS stages (p = 0.012). Any significant relation between clinicopathological features and variation in the final surgical margins was found, just certain tendencies (male patients, persistent tumor, periocular location, and high-risk histopathological tumors required larger margins). Neither patient age or tumor dimension correlated significantly with both number of MMS stages and final surgical margins.

**Study limitations:**

Limitations of this study include its single-center nature with a small sample size, which limits the value of conclusions.

**Conclusion:**

Main factors related to a greater number of MMS stages were periocular location and high-risk histopathological subtype of the tumor.

## Introduction

Mohs Micrographic Surgery (MMS) is a technique that allows intraoperative microscopic control of the surgical margins, being a good treatment option when tissue conservation is required for aesthetic or functional reasons.^1^ Moreover, Basal Cell Carcinomas (BCCs) located in high-risk areas are preferably treated by MMS.^2^ A recent Italian study evaluated the recurrence rate of head and neck high-risk BCCs comparing MMS with conventional surgical excision and confirmed the trend already reported in the literature that MMS is the best treatment option for high-risk BCCs arising in the head and neck region or being recurrent, with a recurrence rate of 3.1% with MMS versus 14% with traditional surgery (p < 0.00001).^3^

Previous studies have shown that both tumor and patient characteristics may predict a higher number of stages necessary to achieve clear margins.^4^, ^5^ Aggressive histological subtypes such as micronodular, infiltrative, and morphea form are well-known factors for a higher number of stages and larger clearance margins.^6^ In a very recent study, Cerci et al. concluded that not just micronodular, infiltrative, and morphea form histological subtypes were associated with larger margins but also superficial ones (due to greater subclinical extension); whereas clinically well-defined and small tumors needed smaller margins, with tumors < 6 mm having a clearance rate of 96% with 3-mm margins; preoperative tumor size was a significant predictor of larger margins and number of MMS stages.^7^ Another recent retrospective study evaluated the characteristics of Basal Cell Carcinoma with Large Subclinical Extension (BCC-LSE), a tumor whose extensive spread becomes apparent during the Mohs surgery histopathology review. Of a total of 2044 cases that met the criteria of BCC-LSE (defined as a lesion requiring at least three Mohs stages and a final surgical margin of ≥10 mm), male sex (p = 0.05), Fitzpatrick skin type I (p = 0.002), history of prior BCC (p = 0.003) and subtypes of basosquamous, metatypical, micronodular, infiltrative, morpheaform and sclerosing (p = 0.005) remained significant BCC-LSE predictors.^5^

Although MMS has been shown to be tissue-sparing, few studies correlated margins required for clearance to tumor characteristics.^7^ Recently, Ariza et al. demonstrated that a 4 mm resection margin was enough to completely eradicate the tumor in 99% of cases of primary small (≤6 mm) facial BCCs, even with aggressive histological patterns (micronodular, infiltrative, or morpheaform). Smaller margins of 2 and 3 mm achieved complete excision of the lesion in 73.9% and 94.4% of cases, respectively.^8^

## Objectives

The main objective of this study is to correlate clinicopathological variables of the patient and tumor with the number of MMS stages required for total tumor clearance. As a secondary endpoint, we aim to correlate the same variables with the final surgical margins required.

## Methods

BCCs treated with MMS in a 2-year period, from July 2018 to July 2020, were retrospectively analysed at the Coimbra University Hospital, Coimbra, Portugal. Data were collected prospectively on each procedure and ‘patient’s features (gender, age, and immune status), ‘tumor’s features (location, primary/recurrent/persistent or previously submitted to incomplete excision, size, histologic subtype, presence of perineural invasion) and surgical features (number of surgical stages and overall surgical margins required for achieving a complete excision) were included. Overall surgical margins are defined as the amount of tissue excised beyond the clinical limits of the tumor and are expressed in millimeters (mm).

Tumor location was stratified by risk of recurrence according to NCCN as high, moderate, and low-risk.^2^ Then four major high-risk areas were further analyzed and compared (i.e., periocular, nose, perioral, ear).

BCCs were classified, according to the histologic subtypes, in low-risk (superficial, nodular, trabecular and ""mixed of low-risk"") and high-risk (micronodular, infiltrative, morpheaform, metatypical and ""mixed of high-risk""). When more than one subtype was present, BCC was classified based on the most aggressive, in one of two groups: ""mixed of high risk"" (when at least one aggressive subtype was present) or ""mixed of low risk"" (when all the subtypes were of low risk).

When a tumor presented with either perineural invasion or a high-risk histologic subtype, it was classified as having aggressive histology or high-risk criteria.

**Figure 1:**
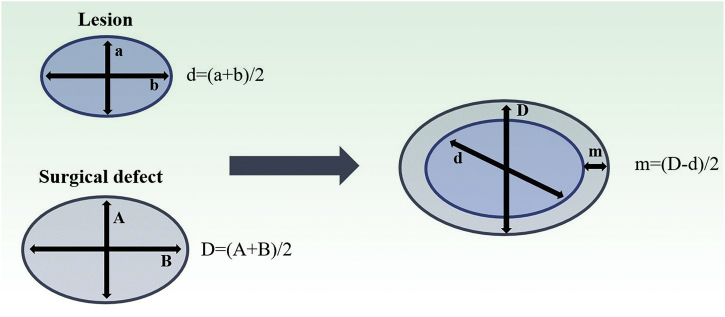
Estimation of final surgical margins based on lesion and defect measurements. Both lesions and defects were measured with length and width. They were averaged. Surgical margin (*m*) is then calculated as half of the difference between lesion and defect diameters (*d*, *D*). Adapted from: Schell AE et al.^11^ Suggested excisional margins for cutaneous malignant lesions based on Mohs micrographic surgery.

Both lesion and final defect after MMS were measured, with length and width reported in millimeters (mm). Then, diameters were calculated as an average of length and width for defect (*D*) and lesion (*d*). The final surgical margin needed (*m*) was calculated as the difference between the final defect diameter (*D*) and the lesion diameter (*d*) (Fig. 1). The present study’s measurements were made by a fixed team of fellowship-trained Mohs surgeons with expertise in this area, using the same method.

### Statistical analysis

Data were analyzed using SPSS version 25 (Armonk, NY: IBM Corp.) After verifying the non-normal distribution of the variables, Mann-Whitney and Kruskal-Wallis tests were performed to compare the number of MMS stages and final margins with other clinicopathological variables, followed by post-hoc analysis for multiple comparisons. The correlation of Spearman was used to correlate quantitative variables. A linear regression model was used to list predictors most associated with a number of surgery times. In the descriptive statistics, qualitative data will be presented in percentage (%) and number (n), and quantitative data with mean and standard deviation when desirable. The significance level was set at p ≤ 0.05.

All patients signed an informed consent giving permission for the use of this data, and the research was conducted according to the local Ethical Committee.

## Results

1) ***Characterization of the population and tumors***

A total of 116 BCCs were included from 113 patients (60 females and 53 males) with a median age of 70 years (range 31‒92) in a mostly non-immunosuppressed population (87.9%). On average, BCCs measured 14.66 mm (range 4‒58 mm), and almost all were located in high-risk areas (94.8%, n = 110), mainly on the nose (n = 77) (Table 1). The majority of BCCs were primary tumours (63.8%, n = 74), 24.1% (n = 28) were recurrent and 12.1% (n = 14) were persistent BCCs (previously submitted to an incomplete excision). Most of them had at least one high risk histologic criteria (74.1%, n = 86) related with an aggressive histologic subtype in 73.3% (n = 85) or perineural invasion in 3.4% (n = 4).

**Table 1:** Distribution of the BCCs treated with MMS by anatomic area and histological subtype.

Variable	Frequency (n)	Percentage (%)
**Location**		
Nose	77	66.4
Perioral	11	9.5
Ear	8	6.9
Periocular^a^	7	6
Frontal	4	3.4
Temporal	3	2.6
Chin	2	1.7
Malar	2	1.7
Extrafacial	2	1.7
**Histological subtype**		
Mixed of high-risk^b^	32	27.6
Infiltrative	26	22.4
Micronodular	16	13.8
Nodular	13	11.2
Mixed of low-risk^b^	12	10.3
Metatypical	6	5.2
Morpheaform	5	4.3
Trabecular	4	3.4
Superficial	2	1.7
**Total**	**116**	**100**

a Periocular tumors include those located in the eyelids and canthus.

b When more than one histological subtype was present, BCC was classified in ""mixed of high risk"" when at least one aggressive subtype was present or ""mixed of low risk"" when all the subtypes were of low risk.

Regarding the high-risk histologic subtypes, the most common was the infiltrative (22.4%, n = 26), followed by micronodular (13.8%, n = 16), metatypical (5.2%, n = 6) and morpheaform (4.3%, n = 5); 32 cases (27.6%) were mixed of high risk (Table 1).

2) ***Relationship between clinicopathological variables and number of MMS stages***

Most of BCCs (61.2%, n = 71) required a single stage of surgery for complete clearance, 31.9% (n = 37) needed two, 5.2% (n = 6) required three and just 0.9% (1) of cases needed four stages, with a mean of 1.45 stages (± 0.64). Table 2 summarizes the relationship of clinicopathological variables with a number of MMS stages.

**Table 2:** Relationship of clinicopathological variables with number of Mohs Micrographic Surgery (MMS) stages and surgical margins required for total tumor excision.

Variable	Mean of MMS stages (±SD)	p-value	Surgical margins in mm (mean ± SD)	p-value
**Sex**		**0.063**		**0.985**
Male	1.56 (± 0.69)		3.25 (± 2.79)	
Female	1.35 (± 0.58)		2.99 (± 1.95)	
**Immunosuppression**		**0.838**		**0.969**
Yes	1.50 (± 0.85)		3.00 (± 2.09)	
No	1.46 (± 0.63)		3.14 (± 2.41)	
**Primary tumour**	1.38 (± 0.51)	**0.279**	2.68 (± 1.56)	**0.234**
**Recurrent tumour**	1.43 (± 0.63)		3.71 (± 3.29)	
**Persistent tumour**	1.86 (± 1.03)		4.27 (± 3.29)	
**Location/subunit**		0.025^a^		**0.811**
Periocular	2.29 (± 0.95)		4.55 (± 3.79)	
Nose	1.42 (± 0.59)		2.80 (± 1.72)	
Perioral	1.36 (± 0.67)		2.93 (± 2.09)	
Ear	1.25 (± 0.46)		2.36 (± 1.07)	
**Aggressive histology**^a^		**0.012**^a^		**0.837**
Yes	1.54 (± 0.68)		3.23 (± 2.60)	
No	1.20 (± 0.41)		2.72 (± 1.31)	
**Histological subtype**		**0.008**^a^		**0.933**
High-risk	**1.55 (**± **0.68)**		**3.23 (**± **2.62)**	
MN	1.44 (± 0.63)		2.87 (± 1.88)	
I	1.60 (± 0.71)		3.75 (± 2.36)	
MO	1.40 (± 0.55)		4.50 (± 3.25)	
MT	1.17 (± 0.41)		1.80 (± 1.28)	
MHR	1.66 (± 0.75)		3.05 (± 3.09)	
Low risk	**1.19 (**± **0.40)**		**2.74 (**± **1.29)**	
S	1.50 (± 0.71)		2.88 (± 0.88)	
N	1.31 (± 0.48)		2.84 (± 1.74)	
T	1.25 (± 0.50)		2.50 (± 0.87)	
MLR	1.00 (± 0.00)			

mm, Millimetres; SD, Standard Deviation; MN, Micronodular;I, Infiltrative; MO, Morpheaform; MT, Metatypical; MHR, Mixed of high risk; S, Superficial; N, Nodular; T, Trabecular; MLR, Mixed of low risk.

The significance level is 0.05

a Aggressive histology: either a high-risk histological subtype or perineural invasion.

Although males required more stages than females (mean 1.56 versus 1.35), this was not statistically significant (p = 0.063). Age and tumor size did not seem to correlate with the number of stages required (*r* = -0.035, p = 0.707; *r* = 0.104, p = 0.271). Even when the authors compare tumors of > 20 mm to those with ≤ 20 mm, no statistically significant differences were found (p = 0.263), although those with *d* > 20 mm required a higher mean of surgical stages (1.55 versus 1.42). Immunosuppressed had a higher mean of MMS stages (1.50 ± 0.85) than immunocompetent (1.46 ± 0.63), though not statistically significant (p = 0.838). Persistent tumors had the highest mean of surgical stages required (1.86), followed by recurrent (1.43) and primary tumors (1.38), even though these differences had no statistical significance (p = 0.279).

Comparing the high-risk subunits, namely periocular area, nose, perioral area, and ear, the authors observed a statistically significant difference in a number of MMS stages required for complete excision (p = 0.025). Table 3 shows which specific groups had a statistically significant difference.

**Table 3:** Influence of location of BCCs in the number of MMS stages required, comparing different high-risk subunits (periocular area, nose, perioral area, and ear).

Location	Mean of MMS stages (± SD)	p-value
**Periocular**	**2.29 (± 0.95)**	
Periocular * ear	2.29 * 1.25	**0.048^a^**
Periocular * nose	2.29 * 1.42	**0.027^a^**
Periocular * perioral	2.29 * 1.36	**0.054^a^**
**Nose**	**1.42 (± 0.59)**	
Nose * ear	1.42 * 1.25	1.000
Nose * perioral	1.42 * 1.36	1.000
**Perioral**	**1.36 (± 0.67)**	
Perioral * ear	1.36 * 1.25	1.000
**Ear**	**1.25 (± 0.46)**	

The significance level is 0.05 (value in **bold^a^**). Significance values have been adjusted by the Bonferroni correction for multiple tests.

Considering histologic aspects, having aggressive histology (either high-risk subtype or perineural invasion) significantly influenced the number of MMS stages (p = 0.012). Moreover, when comparing high-risk and low-risk histological subtypes, the authors also found a statistically significant difference (p = 0.008), with high-risk subtypes requiring more surgical stages (mean of 1.55) than low-risk subtypes (mean of 1.19).

Undertaking a multivariate analysis with a linear regression model, the authors concluded that the number of MMS stages was mostly influenced by the periocular location of the tumor (p < 0.001) and the high-risk histological subtype (p = 0.004). Namely, in periocular tumors, the number of MMS stages increases a mean of 0.89-fold. In high-risk histological subtypes, the number of MMS stages increases a mean of 0.36-fold.

3) ***Correlation of clinicopathological variables with surgical margins***

The mean final surgical defect diameter (*D*) was 20.75 mm (range 6.5–60), with a mean of 3.11 mm for final surgical margins required (*m*).

The authors did not find any significant relation between the clinical or histological features studied and the variation in the final surgical margins (Table 2).

Although males required larger margins for complete tumor removal than females (mean 3.25 mm versus 2.99 mm), this was not statistically significant (p = 0.985). Neither patient’s age nor tumor dimension correlated significantly with final surgical margins (*r* = -0.118, p = 0.227; *r* = 0.029, p = 0.766). Tumors > 20 mm required wider surgical margins (mean 4.22 mm) than those with ≤ 20 mm (mean 2.88 mm), but again without statistical significance (p = 0.166).

Persistent tumors had the highest mean of surgical margins required for complete excision (4.27 mm), followed by recurrent (3.71 mm) and primary tumors (2.68 mm), even though these differences had no statistical significance (p = 0.234).

Tumors located on the periocular area needed the largest margins (mean of 4.55 mm), followed by perioral (mean of 2.93 mm), nose (mean of 2.80 mm) and ear (mean of 2.36 mm), although these differences were not statistically significant (p = 0.811).

Greater surgical margins were needed when there were high-risk histological criteria (mean 3.23 mm versus 2.72 mm), but this was not statistically significant (p = 0.837). Moreover, despite having no statistical significance (p = 0.936), the margins increase with an increasing number of high-risk histological criteria, with a mean of 2.74 mm if there were no histological risk factors, 3.23 mm if there was one, and 3.33 mm when there were two. Comparing high-risk and low-risk histological subtypes, there were differences (high-risk subtypes led to an average margin increase of 3.23 mm and low-risk 2.74 mm), but they were not statistically significant (p = 0.933).

4) ***Surgical aspects***

Debulking was performed in almost all cases (87.1%, n = 101). The majority of surgical defects required a flap for reconstruction (79.3%, n = 92), mainly of advancement type (40.5%, n = 47), followed by transposition (30.2%, n = 35) and rotation (8.6%, n = 10). In 12 patients (10.3%) direct closure was possible; in 7 (6%) a full-thickness skin graft was used, in 1 (0.9%), a compound graft and four defects (3.4%) healed by secondary intention.

The authors found a recurrence rate of 1.7% (n = 2), with a mean time of follow-up of 13.37 months (range 2‒27).

## Discussion

This study aimed to correlate clinicopathologic preoperative features of BCCs with the number of MMS stages (primary endpoint) and the extension of postoperative surgical margins (secondary endpoint) needed for a total clearance of the tumor. Ideally, this could help to identify which tumors should prompt a more carefully surgical procedure and eventually greater initial MMS margins. Regarding the number of MMS stages, the authors have found a significant association with high-risk-histologic subtype, aggressive histology, and localization. However, concerning the final lateral margin, the authors only found a tendency for its increase with certain variables.

The majority of BCCs submitted to MMS in this study were either larger than 1 mm, mostly located on high-risk areas or had at least one high-risk histologic criteria, which is in accordance with the main indications for this type of surgery.^9^

In most cases, only one stage of MMS was needed. Males required more stages than females, approaching to statistical significance (p = 0.063), although not reaching it. Immunosuppression, ‘patient’s age, and tumor size did not influence significatively the number of MMS stages in the present study’s sample. This was not according to other studies, in which preoperative size was a significant predictor of larger margins and number of stages, especially when tumors had > 20 mm.^7^, ^10^, ^11^

Though without statistical significance, persistent tumors had the highest mean of surgical stages required, followed by recurrent and primary tumors. Other studies also showed recurrent tumors to need more surgical stages than primary ones but did not evaluate persistent tumors.^10^, ^11^

Regarding high-risk location, there is a statistically significant difference in a number of stages between tumors located in the periocular region and tumors of the ear, nose, or perioral area, with periocular BCCs requiring more stages of surgery. Although location on high-risk areas was shown to be associated with a higher number of stages in previous studies,^4^, ^5^, ^7^ the specification and comparison between various high-risk areas are more rarely mentioned in the literature.

Having aggressive histology (either high-risk subtype or perineural invasion) significantly influenced the number of MMS stages, and high-risk subtypes (micronodular, infiltrative, morpheaform, metatypical, and ""mixed of high-risk"") required more surgical stages that low-risk subtypes (superficial, nodular, trabecular and ""mixed of low-risk""), which is in line with other studies.^6^ When the authors look at low-risk histological subtypes, superficial BCCs had the highest mean of MMS stages. This is in concordance with a recent study of Cerci et al. that showed that not just micronodular, infiltrative, and morpheaform subtypes, but also superficial ones, were associated with larger margins, concluding that superficial BCCs had a more subclinical extension, leading to larger margins and more surgical stages.^7^ Moreover, in 2013 a group of dermatologists and head and neck surgeons categorized superficial BCCs into a high-risk histologic group because of their irregular growth patterns that led to a higher risk for incomplete excision.^11^

We also evaluated the influence of the same clinicopathological factors in the surgical margins required for tumor clearance, butno statistically significant relation between them was found, only certain tendencies.

One might imagine the tumors and surgical defects as perfectly round-shaped, but in practice, this rarely happens. The configuration of the final defect is usually different from that of the primary tumor, and the increments in length and width are not always harmonious or symmetric, which makes this assessment particularly difficult. Moreover, we must take into account that the measurement technique for excision margins varies largely between authors, being especially difficult to assess it precisely.

Despite this, in the literature, Cerci et al. recently found that BCCs < 6 mm had a clearance rate of 96% with ≤3 mm margins. They also found that superficial, micronodular, infiltrative, and morpheaform histological subtypes were associated with larger margins (mean of 3.1, 3.9, 2.7, and 4 mm, respectively); whereas clinically well-defined tumors were associated with smaller margins (mean of 1.9 mm versus 2.9 mm for ill-defined tumors). Interestingly, however, location in high-risk areas was not significatively associated to larger margins.^7^

In another recent study with 306 small aggressive BCC on the face (defined as tumours with <6 mm on area H and <10 mm on area M), margins required for tumour clearance were also evaluated. They also found that 94.9% of tumors with <6 mm was cleared with 3 mm margins and that a 4 mm resection margin was enough to completely excise the lesion in 99% of cases of primary small facial BCCs with aggressive histological patterns.^8^

In a previous study of Schell et al., including 385 BCCs, mean margins of 2.4 mm and 3.7 mm were found for low-risk and high-risk tumors, respectively, with margins of 4.75 mm and 8 mm achieving 95% clearance.^11^

The authors had a lower rate of relapses than reported in the literature (3.1%‒4.9%),^3^, ^12^, ^13^ which should not be evaluated due to the short follow-up of the present study’s patients at the time of writing.

### Study importance and novelties

The importance of this study resides in its clinical relevance since correlating clinicopathologic preoperative features with both MMS stages and postoperative surgical margins may help to predict which tumors should prompt a more carefully surgical procedure and ideally guide initial margins in MMS. This could help to avoid unnecessary surgical times, reducing not just surgical morbidity but also time and costs. MMS is a highly specialized and very useful surgical technique but it is time-consuming^14^ and requires specialized training, personnel, and equipment, which bring about costs that augment when more surgical times are needed. In the present study, the main factors related to a higher number of MMS stages were the periocular location of the tumor, having aggressive histology, and high-risk histological subtypes. Moreover, in the literature there are very few studies addressing margins required for complete BCC removal using MMS. In this aspect, however, the authors did not find any variable capable of predicting its increase, which can be due to limitations of the study (see next section – study limitations).

In the present study, location in high-risk areas was associated with a higher number of stages, which confirms what was previously reported in the literature. What is new and not previously reported is the statistically significant difference in a number of stages between tumors located in the periocular region and tumors of the ear, nose, or perioral area (all considered high-risk areas), with periocular BCCs requiring more stages of surgery.

Additionally, as far as the authors could investigate in the literature, most studies compare primary and recurrent tumors, but do not evaluate persistent ones (tumors previously submitted to an incomplete excision). In the present study’s sample, persistent tumors had the highest mean of surgical stages required, followed by recurrent and primary tumors, but without statistical significance.

## Study limitations

Limitations of this study include its single-center nature with a small sample size, which limits the value of conclusions.

Moreover, as previously explained, the authors’ method of measuring tumors and surgical defects is not precise and differs from other methods used in the literature (e.g., many authors use the main axis of the lesion rather than the mean of both length and width). This can at least partially explain the difficulties found in establishing statistically significant relations regarding final surgical margins. In the future, it will be useful to find a more accurate and universal method for these measurements. The measures should ideally include the depth of the lesion, giving it a Three-Dimensional (3D) view closer to the real.

## Conclusion

In this study, the main factors related to a higher number of MMS stages were the periocular location of the tumor and high-risk histological subtypes (micronodular, infiltrative, morpheaform, metatypical and "mixed of high-risk"). None of the other studied factors showed to statistically influence the number of MMS stages. We were not able to find any variable capable of predicting the increase in the final margin required for complete excision, which could be due to the relatively small sample size or technical limitation of measurement of margin increment. Larger prospective studies would be beneficial to corroborate these results.

## Financial support

None declared.

## Authors’ contributions

Joana Calvão: Approval of the final version of the manuscript; critical literature review; data collection, analysis, and interpretation; effective participation in research orientation; intellectual participation in propaedeutic and/or therapeutic management of studied cases; critical manuscript review; preparation and writing of the manuscript; statistical analysis; study conception and planning.

André Pinho: Approval of the final version of the manuscript; data collection, analysis, and interpretation; effective participation in research orientation; intellectual participation in propaedeutic and/or therapeutic management of studied cases; critical manuscript review; statistical analysis; study conception and planning.

Ana Brinca: Approval of the final version of the manuscript; data collection, analysis, and interpretation; intellectual participation in propaedeutic and/or therapeutic management of studied cases.

Ricardo Vieira: Approval of the final version of the manuscript; data collection, analysis, and interpretation; effective participation in research orientation; intellectual participation in propaedeutic and/or therapeutic management of studied cases; critical manuscript review; study conception and planning.

## Conflicts of interest

None declared.
